# Assessment of Reproducibility and Agreement of the IDEAL Classification for Distal Radius Fractures

**DOI:** 10.1055/s-0044-1792121

**Published:** 2024-12-21

**Authors:** João Victor da Rocha Lima, Lucas Araújo Silva, Antonio Guilherme Chagas Silva Feitosa, Rafael Lima Medeiros, Luis Fernando Martins Carvalho, Bruno Wilson da Silva Moura

**Affiliations:** 1 Unidade de Traumato-Ortopedia, Hospital Universitário da Universidade Federal do Piauí (HUUFPI), Teresina, PI, Brasil

**Keywords:** classification, radius fractures, reproducibility of results

## Abstract

**Objective** To analyze the reproducibility and intra- and interobserver agreement of the IDEAL classification for distal radius fractures.

**Methods** This qualitative, analytical study evaluated 50 pairs of radiographs in two views of patients with distal radius fractures. There were ten observers with different levels of orthopedic training who assessed the radiographs in three different evaluations. The results underwent the Cohen and Fleiss Kappa tests to determine intra- and interobserver agreement levels. Statistical calculations used Excel and SPSS, version 26.0.

**Results** The Cohen Kappa index values for intraobserver evaluation indicated poor to little agreement (-0.177–0.259), with statistical significance in only one instance. The Fleiss Kappa index values revealed little agreement among the resident group (0.277–0.383) with statistical significance, poor to little agreement among the general orthopedists (0.114–0.225) with statistical significance in most instances, and moderate agreement among hand surgeons (0.449–0.533) with statistical significance.

**Conclusion** The IDEAL classification had interobserver agreement levels ranging from poor to moderate, influenced by the physicians' training level. Other intraobserver agreement levels ranged from poor to little agreement but without statistical significance.

## Introduction

Distal radius fractures are extremely prevalent, accounting for 16% of body and 74% of forearm fractures. They present a bimodal distribution, affecting adolescents/young adults (high-energy trauma) and the elderly (low-energy trauma). The most common mechanism of injury is a fall to the ground with the wrist in hyperextension. 1 2 3 4

Despite the high prevalence, there has never been much consensus in the literature regarding the best classification for distal radius fractures. The first concepts began before the advent of radiography, with the description from Colles for fractures with dorsal displacement in 1814. In 1951, Gartland and Werley proposed the first classification for fractures of the distal radius, followed by Frykman in 1967, the AO group's from Muller in 1986, Fernandez's in 1991, the Universal one from Cooney in 1993, and, most recently, the IDEAL classification from the Division of Hand Surgery from the Universidade Federal de São Paulo (UNIFESP), in 2013. 1 3 5

The IDEAL classification relies on five parameters (two epidemiological and three radiographic), namely: age (younger or older than 60), energy of the trauma resulting in the fracture, fragment displacement (presence or absence), joint incongruity (incongruence or separation > 2 millimeters), and associated injuries (presence or absence). Each parameter scores zero or one point and their sum gives the fracture classification, with types I (0–1 points), II (2–3), or III (4–5). Each type suggests a treatment and prognosis of the injury. 1

Previous studies show low to moderate levels of intra- and interobserver agreement for the oldest classifications available in the literature, such as the Frykman, Fernandez, and AO. The Universal and IDEAL classifications presented better results compared to the previous ones. 1 2 Those with more subtypes and divisions presented lower interobserver agreement, which may raise issues concerning intraobserver agreement due to the longer time needed to get used to the instrument. 1 5 6 7 8 9 10 11 12

Because there are several classifications for fractures of the distal end of the radius, it is essential to define the best one in studies such as this one, assessing their reproducibility and reliability. This study aims to analyze the reproducibility and intra- and interobserver agreement of the IDEAL classification for distal radius fractures, and to determine the influence of the observers' training level.

## Materials and Methods

This qualitative, analytical, retrospective, and direct documentation study evaluated radiographs of patients with distal radius fractures by observers with different experience levels in traumatology. The research occurred at a University Hospital, which provided the radiographs and allowed interviews for data collection from November to December 2022.

The Giraudeau and Mary method 13 for sample determination, per the expected level of agreement, the number of evaluators, and the confidence interval (CI) estimated several minimum samples. Table 1 shows that an expected Kappa of 0.70 and a 90% confidence interval requires a minimum of 41 samples. 14 We obtained 50 pairs of radiographs (anteroposterior and lateral views) showing distal radius fractures from the electronic medical records of patients treated at this university hospital from 2019 to 2022.

**Table 1:** Sample size estimation using the intraclass correlation coefficient based on Giraudeau and Mary

		Number of participants required for a 95% CI at three confidence levels		
Number of observers	Expected ICC	± 0.05	± 0.10	± 0.15
2	0.9	56	14	4
	0.8	200	50	13
	0.7	400	100	25
	0.6	630	158	40
	0.5	865	217	55
4	0.9	36	9	3
	0.8	119	30	8
	0.7	222	56	14
	0.6	322	81	21
	0.5	401	101	26
6	0.9	31	8	2
	0.8	103	26	7
	0.7	187	47	12
	0.6	263	66	17
	0.5	314	79	20
10+	0.9	29	8	2
	0.8	92	23	6
	0.7	164	41	11
	0.6	224	56	14
	0.5	259	65	17

**Abbreviations:** CI, confidence interval; ICC, intraclass correlation coefficient.

**Note:** Adapted from Karanicolas et al. 14

The inclusion criteria were patients whose medical records had the International Classification of Diseases (ICD) for distal radius fractures (S52.5) and who received treatment at the University Hospital. The exclusion criteria were patients who had undergone any type of treatment, surgical or otherwise, for a distal radius fracture before the radiograph, and with no imaging of distal radius fracture available in their medical records.

There were three orthopedic specialists in hand surgery, four general orthopedic surgeons from the orthopedic service of the University Hospital, and three orthopedic residents, one from each year, from the University Hospital, who participated in the study as observers. They evaluated the radiographs to classify them according to the IDEAL method. The evaluation occurred three times, with an interval of 15.3 ± 4.34 days.

We tabulated the results from the observers' assessments in Microsoft Excel 2019 (Microsoft Corp., Redmond, WA, USA) and performed the Cohen and Fleiss Kappa tests for intra- and interobserver assessment, respectively, using the Statistical Package Social Sciences (SPSS, IBM Corp., Armonk, NY, USA), version 26.0, for statistical analysis. 15 Interobserver agreement assessment tables showed the Kappa index measurement for each observer class (residents, general orthopedists, and hand surgeons) for the three separate assessments. Intraobserver agreement assessment tables compared each assessment with the other two and determined the presence of upper and lower limit values for a 90% confidence interval (CI).

Kappa values with *p* < 0.1 were considered significant. The interpretation of results used the method proposed by Landis and Koch, in which values lower than and up to zero indicate poor agreement, with little agreement from 0 to 0.2, reasonable from 0.2 to 0.4, moderate from 0.4 to 0.6, substantial from 0.6 to 0.8, and excellent or virtually perfect agreement from 0.8 to 1. 16

The Research Ethics Committee approved this research under the CAAE number 63490322.8.0000.8050 and opinion number 5,726,415.

## Results

The Cohen Kappa indexes for intraobserver agreement ( Table 2 ) were 0.259 (poor agreement) in one instance (CM1 T1 x T2), with statistical significance ( *p* = 0.021), and 0.140 or lower (poor agreement) in all others, with no statistical significance in any case ( *p* > 0.1).

**Table 2:** The Cohen Kappa index and their *p* -values for the intraobserver agreement tests

	T1 x T2		T2 x T3		T1 x T3	
	κ	*p*	κ	*p*	κ	*p*
R1	0.091	0.384	0.028	0.786	0.049	0.660
R2	0.140	0.174	-0.078	0.419	-0.026	0.805
R3	-0.151	0.139	-0.043	0.673	0.015	0.885
GO1	-0.022	0.838	-0.053	0.624	-0.017	0.872
GO2	0.009	0.940	-0.177	0.159	0.006	0.963
GO3	-0.121	0.255	0.054	0.646	-0.069	0.570
GO4	0.108	0.352	-0.032	0.779	-0.029	0.797
HS1	0.259	0.021	-0.078	0.463	-0.009	0.933
HS2	0.138	0.178	0.028	0.791	0.042	0.683
HS3	0.028	0.791	-0.053	0.646	0.006	0.956

**Abbreviation:** HS, hand surgeon; GO, general orthopedist; R, resident in orthopedics and traumatology.

Table 3 shows the Fleiss Kappa indexes for interobserver agreement in the resident group ranged from 0.277 to 0.383 in the three assessments, with statistical significance. The CIs do not contain the parameter value and *p* ≤ 0.008.

**Table 3:** The Fleiss Kappa indexes for interobserver agreement between residents in orthopedics and traumatology for each assessment and IDEAL classification type

	κ index	*p*	90% CI lower limit	90% CI upper limit
κ T1	0.305	< 0.001	0.206	0.404
Type 1	0.349	< 0.001	0.214	0.483
Type 2	0.215	0.008	0.081	0.350
Type 3	0.386	< 0.001	0.252	0.521
κ T2	0.383	< 0.001	0.282	0.483
Type 1	0.435	< 0.001	0.301	0.569
Type 2	0.302	< 0.001	0.167	0.436
Type 3	0.452	< 0.001	0.317	0.586
κ T3	0.277	< 0.001	0.175	0.378
Type 1	0.308	< 0.001	0.173	0.442
Type 2	0.250	0.002	0.116	0.384
Type 3	0.292	< 0.001	0.157	0.426

**Abbreviation:** CI, confidence interval.

Table 4 shows the Fleiss Kappa indexes for the general orthopedists ranged from 0.114 to 0.225, with statistical significance. The CIs do not contain the parameter value and *p* ≤ 0.008.

**Table 4:** Interobserver Fleiss Kappa index of orthopedists for each assessment and IDEAL classification type

	κ index	*p*	90% CI lower limit	90% CI upper limit
κ T1	0.186	< 0.001	0.115	0.258
Type 1	0.472	< 0.001	0.377	0.567
Type 2	0.112	0.053	0.017	0.207
Type 3	0.065	0.261	-0.030	0.160
κ T2	0.114	0.008	0.043	0.184
Type 1	0.330	< 0.001	0.235	0.425
Type 2	0.011	0.849	-0.084	0.106
Type 3	0.090	0.119	-0.005	0.185
κ T3	0.225	< 0.001	0.154	0.295
Type 1	0.359	< 0.001	0.335	0.454
Type 2	0.148	< 0.001	0.361	0.243
Type 3	0.223	< 0.001	0.485	0.318

**Abbreviation:** CI, confidence interval.

Table 5 shows the Fleiss Kappa indexes for the hand surgeons ranged from 0.449 to 0.553, all with statistical significance. Results for each classification type were higher for type III in all evaluations when compared with types I and II, but all assessments had excellent significance levels. The CIs do not contain the parameter value and *p* < 0.001).

**Table 5:** Interobserver Fleiss Kappa index of hand surgeons for each assessment and IDEAL classification type

	κ index	*p*	90% CI lower limit	90% CI upper limit
Fleiss' κ T1	0.533	< 0.001	0.430	0.637
Type 1	0.469	< 0.001	0.335	0.604
Type 2	0.495	< 0.001	0.361	0.629
Type 3	0.620	< 0.001	0.485	0.754
Fleiss' κ T2	0.449	< 0.001	0.347	0.550
Type 1	0.365	< 0.001	0.231	0.500
Type 2	0.430	< 0.001	0.296	0.564
Type 3	0.525	< 0.001	0.391	0.659
Fleiss' κ T3	0.531	< 0.001	0.430	0.631
Type 1	0.627	< 0.001	0.493	0.761
Type 2	0.470	< 0.001	0.336	0.604
Type 3	0.542	< 0.001	0.407	0.676

**Abbreviation:** CI, Confidence interval.

## Discussion

Distal radius fractures are prevalent, requiring a complete understanding of the potential fracture patterns' complexity and broadening its scope to other factors impacting their prognosis. 10 12 The IDEAL classification meets these requirements, as it includes age and trauma energy in its parameters.

The limitations of this study included the low number of observers for each category and the absence of hand surgery residents.

We observed a tendency towards little or no agreement, or even disagreement, in the intraobserver evaluation, with most cases not presenting considerable statistical significance. This finding is inconsistent with the literature, in which most studies detected moderate to high agreements. 1 3 4 5

The degree of interobserver agreement measured by the Fleiss Kappa index had more statistical solidity than the Cohen one in analyzing intraobserver agreement. Observers had difficulty agreeing on the intermediate type of classification, and it was easier on the extremes. Given the low or even absent agreement between orthopedists and residents, we can infer that the difference in training level apparently did not imply a higher level of agreement for the group with more experience in orthopedics, as we observed an even greater agreement among residents. Andersen et al. 17 and Belloti et al. 18 reported no influence from the observers' experience level, which is consistent with our findings since our less experienced observers had higher agreement levels and better statistical significance when compared with more experienced ones.

In contrast, hand surgeons obtained the best levels of interobserver agreement among the three observer groups, with moderate levels in all three assessments. Results from hand surgeons allow us to infer that the additional specific training enabled them to obtain more concordant results than the other groups. In this scenario, differing from Illarramendi et al., 8 Andersen et al., 17 Jayakumar et al., 3 and Belloti et al., 18 the hand surgeons' additional experience was the main factor for the best interobserver agreement levels in this study.

The general objective of classifications is to provide a tool to accurately classify a fracture into a type to guide its treatment and define a prognosis. They also allow effective communication between professionals from different backgrounds. 4 Although we did not detect high levels (> 0.8) of intra- and interobserver agreement in this or other studies in the literature, 1 3 4 5 6 7 8 it seems that fulfilling this objective is possible.

## Conclusion

This study found interobserver agreement levels ranging from poor to moderate, demonstrating that the training level only influenced the results from hand surgeons, with no significant difference between residents and orthopedists. In conclusion, the classification proved to be, to a certain extent, irreproducible and inconsistent.

Nevertheless, it is essential to perform further studies of this type, either with this or other classifications, to provide increasingly solid scientific evidence and allow the choice of the best classification.
