# Supplementary material for: Assessment of Reproducibility and Agreement of the IDEAL Classification for Distal Radius Fractures
Source: Rev Bras Ortop (Sao Paulo). 2024 Dec 21;59(6):e901–6. doi: 10.1055/s-0044-1792121 (PMC11663075; doi:10.1055/s-0044-1792121)
Supplement: Related texts

# Research article: Avaliação da reprodutibilidade e concordância da classificação IDEAL para fraturas de extremidade distal de rádio

**Keywords:** classificação, fraturas do rádio, reprodutibilidade dos testes

## Abstract

**Objetivo** Analisar a reprodutibilidade e concordância intra- e interobservadores da classificação IDEAL para fraturas de extremidade distal do rádio.

**Métodos** Estudo qualitativo, analítico, realizado pela avaliação de 50 pares de radiografias em duas incidências de pacientes com fratura da extremidade distal do rádio. As radiografias foram avaliadas por dez observadores de diferentes níveis de treinamento em ortopedia em três avaliações distintas. Os resultados foram submetidos aos testes Kappa de Cohen e Fleiss para obtenção dos níveis de concordância intra- e interobservadores. Foram utilizados os programas Excel e SPSS, versão 26.0, para cálculos estatísticos.

**Resultados** Os valores do índice Kappa de Cohen para avaliação intraobservadores indicaram pobre a pouca concordância (-0,177–0,259), com significância estatística em apenas uma instância. Os valores do índice Kappa de Fleiss revelaram pouca concordância entre o grupo de residentes (0,277–0,383) com significância estatística; concordância pobre a pouca entre o grupo de ortopedistas gerais (0,114–0,225) com significância estatística na maioria das instâncias; e concordância moderada entre o grupo de cirurgiões de mão (0,449–0,533) com significância estatística.

**Conclusão** A classificação IDEAL obteve níveis de concordância interobservadores variando entre pobre e moderado, com influência do nível de treinamento dos profissionais. Os outros níveis de concordância intraobservadores variaram de pobre a pouca concordância, mas sem significância estatística.

## Introdução

As fraturas da extremidade distal do rádio são extremamente prevalentes, representando 16% das fraturas do corpo e 74% do antebraço, com distribuição bimodal, acometendo adolescentes/adultos jovens (traumas de alta energia) e idosos (traumas de baixa energia). O mecanismo de trauma mais comum é a queda ao solo com punho em hiperextensão. 1 2 3 4

Apesar da prevalência elevada, nunca houve bastante consenso na literatura em relação a uma classificação de escolha para fraturas de extremidade distal do rádio. Os primeiros conceitos se iniciaram antes do advento da radiografia, com a descrição dada por Colles para a fratura com desvio dorsal em 1814. Em 1951, Gartland e Werley propuseram a primeira classificação para fraturas da extremidade distal de rádio, seguidos por Frykman em 1967, pelo grupo AO proposta por Muller em 1986, a de Fernandez em 1991, a Universal proposta por Cooney em 1993, chegando à classificação IDEAL, proposta pela Divisão de Cirurgia da Mão da Universidade Federal de São Paulo (UNIFESP), em 2013. 1 3 5

A classificação IDEAL se baseia em cinco parâmetros (dois epidemiológicos e três radiográficos), sendo eles: idade (maior ou menor que 60 anos), energia do trauma que ocasionou a fratura, desvio dos fragmentos (presença ou não), incongruência articular (incongruência ou afastamento > 2 milímetros) e lesões associadas (presença ou ausência). Cada um dos parâmetros pontua zero ou um e o somatório deles dá a classificação da fratura, sendo dos tipos I (0–1 pontos), II (2–3) ou III (4–5). Cada tipo na descrição sugere um tratamento e o prognóstico da lesão. 1

Estudos anteriores evidenciam baixos a moderados níveis de concordância intra e interobservadores para as classificações mais antigas disponíveis na literatura, como as classificações de Frykman, Fernandez e AO. As classificações Universal e IDEAL apresentaram resultados melhores em comparação às demais. 1 2 Verificou-se que aquelas com mais subtipos e divisões apresentaram menor concordância interobservadores, podendo suscitar problemas em relação à concordância intraobservador pelo maior tempo necessário para se habituar ao uso do instrumento. 1 5 6 7 8 9 10 11 12

Em razão da existência de várias classificações para as fraturas de extremidade distal do rádio, se torna importante definir a melhor, através de estudos como este, que avaliam sua reprodutibilidade e confiabilidade. Este estudo tem por objetivo analisar a reprodutibilidade e concordância intra- e interobservadores da classificação IDEAL para fraturas de extremidade distal do rádio, assim como verificar a influência do nível de treinamento dos observadores.

## Materiais e Métodos

Trata-se de um estudo qualitativo, analítico, retrospectivo e de documentação direta, realizado a partir da avaliação de radiografias de pacientes acometidos por fratura de extremidade distal de rádio por observadores de diferentes níveis de experiência em traumatologia. A pesquisa foi realizada nas dependências de um Hospital Universitário, onde foram obtidas as radiografias para realização do estudo e conduzidas as entrevistas para obtenção dos dados, nos meses de novembro e dezembro de 2022.

De acordo com o método de Giraudeau e Mary 13 para determinação da amostra de acordo com o nível de concordância esperado, a quantidade de avaliadores e o intervalo de confiança (IC), diversas quantidades mínimas para a amostra são esperadas. Conforme demonstrado na Tabela 1 , para um Kappa esperado de 0.70 e um intervalo de confiança de 90%, um mínimo de 41 amostras são necessárias. 14 Foram obtidos 50 pares de radiografias (incidências anteroposterior e lateral) que evidenciem fraturas de extremidade distal de rádio dentre os prontuários eletrônicos dos pacientes atendidos neste Hospital Universitário entre os anos de 2019 e 2022.

**Tabela 1:** Estimativa do tamanho da amostra com uso do coeficiente de correlação intraclasse baseado em Giraudeau e Mary

		Número de participantes requeridos para um IC 95% em três níveis de confiança		
Número de avaliadores	ICC esperado	± 0.05	± 0.10	± 0.15
2	0,9	56	14	4
	0,8	200	50	13
	0,7	400	100	25
	0,6	630	158	40
	0,5	865	217	55
4	0,9	36	9	3
	0,8	119	30	8
	0,7	222	56	14
	0,6	322	81	21
	0,5	401	101	26
6	0,9	31	8	2
	0,8	103	26	7
	0,7	187	47	12
	0,6	263	66	17
	0,5	314	79	20
10+	0,9	29	8	2
	0,8	92	23	6
	0,7	164	41	11
	0,6	224	56	14
	0,5	259	65	17

**Abreviações:** IC, intervalo de confiança; ICC, coeficiente de correlação intraclasse.

**Nota:** Adaptado de Karanicolas et al. 14

Os critérios de inclusão foram: pacientes cujos prontuários contenham o CID de fratura de extremidade distal do rádio (S52.5); pacientes que tenham sido atendidos no Hospital Universitário. Os critérios de exclusão foram: pacientes que tenham sido submetidos a algum tipo de tratamento, cirúrgico ou não, para a fratura de extremidade distal do rádio antes da obtenção da radiografia; pacientes que não apresentem em seus prontuários a disponibilidade do exame de imagem da fratura de extremidade distal do rádio.

Foram convidados a participar da pesquisa como observadores três ortopedistas especialistas em cirurgia da mão, quatro ortopedistas gerais pertencentes ao serviço de ortopedia do Hospital Universitário e três residentes de ortopedia, um de cada ano, do Hospital Universitário. Avaliaram as radiografias para classificá-las de acordo com a classificação IDEAL. A avaliação se deu em três momentos diferentes, separados por 15,3 ± 4,34 dias.

Os resultados obtidos com as avaliações dos observadores foram tabulados utilizando o Microsoft Excel 2019 (Microsoft Corp., Redmond, WA, EUA) e submetidos aos testes Kappa de Cohen e Fleiss para avaliação intra- e interobservadores, respectivamente, através do software Statistical Package Social Sciences (SPSS, IBM Corp., Armonk, NY, USA), versão 26.0, para análises estatísticas. 15 Obtiveram-se as tabelas de avaliação de concordância interobservadores evidenciando a medida do índice de Kappa para cada classe de avaliadores (residentes, ortopedistas gerais e cirurgiões de mão) para cada uma das três avaliações. As tabelas de avaliação de concordância intraobservador, comparando cada uma das avaliações com as outras duas, bem como a presença dos valores limites superior e inferior para um IC de 90%.

Foram considerados significativos os valores de Kappa com *p* < 0,1. A interpretação dos resultados seguindo o entendimento proposto por Landis e Koch, no qual valores menores que e até zero indicavam concordância pobre, pouca entre 0 e 0,2, razoável entre 0,2 e 0,4, moderada entre 0,4 e 0,6, substancial entre 0,6 e 0,8, e excelente ou quase perfeita entre 0,8 e 1. 16

Esta pesquisa foi autorizada pelo Comitê de Ética em Pesquisa sob o número CAAE: 63490322.8.0000.8050, parecer de n° 5.726.415.

## Resultados

Os valores dos índices Kappa de Cohen obtidos para a concordância intraobservadores ( Tabela 2 ) foram de 0,259 (pouca concordância) em uma instância (CM1 T1 x T2), com significância estatística ( *p* = 0.021), e valores de 0,140 ou menos (concordância pobre) em todas as outras, sem significância estatística em nenhuma delas ( *p* > 0,1).

**Tabela 2:** Índices de Kappa de Cohen e valores-p dos mesmos nos testes de concordância intraobservadores

	T1 x T2		T2 x T3		T1 x T3	
	κ	*p*	κ	*p*	κ	*p*
R1	0,091	0,384	0,028	0,786	0,049	0,660
R2	0,140	0,174	-0,078	0,419	-0,026	0,805
R3	-0,151	0,139	-0,043	0,673	0,015	0,885
OG1	-0,022	0,838	-0,053	0,624	-0,017	0,872
OG2	0,009	0,940	-0,177	0,159	0,006	0,963
OG3	-0,121	0,255	0,054	0,646	-0,069	0,570
OG4	0,108	0,352	-0,032	0,779	-0,029	0,797
CM1	0,259	0,021	-0,078	0,463	-0,009	0,933
CM2	0,138	0,178	0,028	0,791	0,042	0,683
CM3	0,028	0,791	-0,053	0,646	0,006	0,956

**Abreviações:** CM, cirurgião de mão; OG, ortopedista geral; R, residente de ortopedia e traumatologia.

Na Tabela 3 , observa-se índices de Kappa de Fleiss para concordância interobservadores do grupo de Residentes variando entre 0,277 e 0,383 nas três avaliações, com significância estatística. Os ICs não contém o valor-parâmetro e *p* ≤ 0,008.

**Tabela 3:** Índices de Kappa de Fleiss para concordância interobservadores do grupo de Residentes em Ortopedia e Traumatologia de cada avaliação e para cada tipo descrito na classificação IDEAL

	Índice κ	*p*	Limite inferior IC 90%	Limite superior IC 90%
κ de Fleiss T1	0,305	< 0,001	0,206	0,404
Tipo 1	0,349	< 0,001	0,214	0,483
Tipo 2	0,215	0,008	0,081	0,350
Tipo 3	0,386	< 0,001	0,252	0,521
κ de Fleiss T2	0,383	< 0,001	0,282	0,483
Tipo 1	0,435	< 0,001	0,301	0,569
Tipo 2	0,302	< 0,001	0,167	0,436
Tipo 3	0,452	< 0,001	0,317	0,586
κ de Fleiss T3	0,277	< 0,001	0,175	0,378
Tipo 1	0,308	< 0,001	0,173	0,442
Tipo 2	0,250	0,002	0,116	0,384
Tipo 3	0,292	< 0,001	0,157	0,426

**Abreviação:** IC, intervalo de confiança.

A Tabela 4 traz índices de Kappa de Fleiss do grupo de Ortopedistas Gerais com valores entre 0,114 e 0,225, com significância estatística. Os ICs não contém o valor-parâmetro e *p* ≤ 0,008.

**Tabela 4:** Índices de Kappa de Fleiss interobservadores do grupo de Ortopedistas de cada avaliação e para cada tipo descrito na classificação IDEAL

	Índice κ	*p*	Limite inferior IC 90%	Limite superior IC 90%
κ de Fleiss T1	0,186	< 0,001	0,115	0,258
Tipo 1	0,472	< 0,001	0,377	0,567
Tipo 2	0,112	0,053	0,017	0,207
Tipo 3	0,065	0,261	-0,030	0,160
κ de Fleiss T2	0,114	0,008	0,043	0,184
Tipo 1	0,330	< 0,001	0,235	0,425
Tipo 2	0,011	0,849	-0,084	0,106
Tipo 3	0,090	0,119	-0,005	0,185
κ de Fleiss T3	0,225	< 0,001	0,154	0,295
Tipo 1	0,359	< 0,001	0,335	0,454
Tipo 2	0,148	< 0,001	0,361	0,243
Tipo 3	0,223	< 0,001	0,485	0,318

**Abreviação:** IC, intervalo de confiança.

A Tabela 5 traz índices de Kappa de Fleiss do grupo de Cirurgiões de Mão apresentando valores entre 0,449 e 0,553, todos com significância estatística. Os resultados observados para cada tipo da classificação apresentam valores maiores para o tipo III em todas as avaliações em comparação com os tipos I e II, mas todas as avaliações apresentaram excelentes níveis de significância. Os ICs não contém o valor-parâmetro e *p* < 0,001.

**Tabela 5:** Índices de Kappa de Fleiss interobservadores do grupo de Cirurgiões da Mão de cada avaliação e para cada tipo descrito na classificação IDEAL

	Índice κ	*p*	Limite inferior IC 90%	Limite superior IC 90%
κ de Fleiss T1	0,533	< 0,001	0,430	0,637
Tipo 1	0,469	< 0,001	0,335	0,604
Tipo 2	0,495	< 0,001	0,361	0,629
Tipo 3	0,620	< 0,001	0,485	0,754
κ de Fleiss T2	0,449	< 0,001	0,347	0,550
Tipo 1	0,365	< 0,001	0,231	0,500
Tipo 2	0,430	< 0,001	0,296	0,564
Tipo 3	0,525	< 0,001	0,391	0,659
κ de Fleiss T3	0,531	< 0,001	0,430	0,631
Tipo 1	0,627	< 0,001	0,493	0,761
Tipo 2	0,470	< 0,001	0,336	0,604
Tipo 3	0,542	< 0,001	0,407	0,676

**Abreviação:** IC, intervalo de confiança.

## Discussão

As fraturas da extremidade distal do rádio, sendo um diagnóstico tão prevalente, necessitam de uma total compreensão de toda a complexidade dos padrões de fratura que podem surgir e ampliação de seu escopo para outros fatores que implicam sobre o prognóstico da patologia. 10 12 A classificação IDEAL cumpre este aspecto, pois envolve idade e energia do trauma em seus parâmetros.

Entre as limitações deste estudo, pode-se citar o baixo número de avaliadores para cada categoria, além da ausência da categoria residentes em cirurgia da mão.

Observou-se primariamente uma tendência à resultados com pouca ou nenhuma concordância, ou até mesmo discordância na avaliação intraobservadores, com a maioria das instâncias não apresentando significância estatística considerável, achado que difere do que foi encontrado na literatura, com a maioria dos estudos obtendo resultados com concordâncias de moderada a alta. 1 3 4 5

O grau de concordância interobservadores medido pelo índice Kappa de Fleiss mostrou um resultado com mais solidez estatística do que o de Cohen na análise de concordância intraobservadores. Pode-se verificar uma dificuldade dos observadores em concordar no tipo intermediário da classificação, sendo mais fácil nos extremos. Dada a baixa ou até ausente concordância entre os ortopedistas em relação aos residentes, é possível inferir que a diferença no nível de treinamento aparentemente não implicou em um maior nível de concordância para o grupo com mais experiência em ortopedia, observando-se até mesmo uma maior concordância entre os residentes. Nos estudos de Andersen et al. 17 e Belloti et al. 18 não houve influência do nível de experiência do observador sobre os resultados obtidos, o que corrobora com os achados deste estudo, visto que os observadores menos experientes obtiveram resultados com maiores níveis de concordância e melhores níveis de significância estatística quando comparados aos mais experientes.

O grupo dos cirurgiões da mão, por outro lado, obteve os melhores níveis de concordância interobservadores entre os três grupos avaliados, com níveis moderados em todas as três instâncias da pesquisa. Com os resultados obtidos pelo grupo dos cirurgiões de mão, infere-se que o treinamento adicional específico possibilitou ao grupo obter resultados mais concordantes entre si quando comparados com os demais. Neste cenário, diferindo do que foi proposto por Illarramendi et al., 8 Andersen et al., 17 Jayakumar et al. 3 e Belloti et al., 18 a experiência adicional do grupo de cirurgiões de mão foi o fator preponderante para a obtenção dos melhores níveis de concordância interobservadores deste estudo.

O objetivo das classificações, de forma geral, é fornecer uma ferramenta que consiga enquadrar a fratura em um tipo ou classe de forma acurada e guiar o tratamento dessa fratura enquanto define prognóstico para a mesma, e também possibilite a comunicação efetiva entre profissionais das mais diferentes origens. 4 Apesar de não se verificar níveis elevados (> 0,8) de concordância intra- e interobservadores para as classificações neste ou nos demais estudos presentes na literatura, 1 3 4 5 6 7 8 verifica-se que há a possibilidade de cumprir com o objetivo supracitado.

## Conclusão

O presente estudo encontrou níveis de concordância interobservadores variando entre pobre e moderado, além de demonstrar que o nível de treinamento só interferiu nos resultados no grupo dos cirurgiões de mão, sem diferença significativa entre residentes e ortopedistas. Em conclusão, a classificação se mostrou, até certo ponto, irreprodutível e não concordante.

Não obstante, é essencial que sejam realizados novos estudos deste tipo, com esta ou outras classificações, com o objetivo de fornecer cada vez mais solidez às evidências científicas e permitir a escolha da melhor classificação.
